# Notch Signaling in the Regulation of Hematopoietic Stem Cell

**DOI:** 10.1007/s40778-017-0090-8

**Published:** 2017-07-10

**Authors:** Fabio Pereira Lampreia, Joana Gonçalves Carmelo, Fernando Anjos-Afonso

**Affiliations:** 1 Haematopoietic Signalling Group, European Cancer Stem Cell Research Institute, School of Biosciences, Cardiff University, Hadyn Ellis Building, Maindy Road, Cardiff, CF24 4HQ UK; 2 Department of Bioengineering and iBB-Institute for Bioengineering and Biosciences, Instituto Superior Técnico, Universidade de Lisboa, Av. Rovisco Pais, 1049–001 Lisbon, Portugal

**Keywords:** Notch signaling, HSC, Niche, MPD

## Abstract

**Purpose of Review:**

Understanding the signaling pathways that govern hematopoietic stem and progenitor cells (HSPCs) is fundamental to uncover their regulation and how this is skewed in hematological malignancies. Whether Notch is necessary for the regulation of mammalian HSPCs is still unclear. We therefore critically review the current literature on the role of Notch in HSPCs.

**Recent Findings:**

HSPCs have shown different requirements for Notch signals in vitro and in vivo and at different stages of differentiation. Additionally, bone marrow niche cells activate Notch signaling in HSPCs enhancing their regenerative and self-renewal capacity.

**Summary:**

Despite the controversy, adequate levels of Notch signaling appear necessary to avoid the development of hematological malignancies. Contrary to early studies, recent research suggests that Notch signaling may play a role in homeostatic and regenerative hematopoiesis but further investigation is necessary to understand how it is regulated by the different ligand/receptor pairings and the molecular mechanisms that are triggered.

## Introduction: Brief Description of Notch Signaling

Detailed reviews on the Notch pathway can be found elsewhere [1, 2]. Briefly, in mammals, there are four Notch receptors (Notch1, 2, 3, and 4*)* and five structurally related, single-pass membrane Notch ligands (Delta-like1, 3, and 4 and Jagged1 and 2). The canonical Notch signaling is activated through ligand-mediated interaction that instigates the first cleavage of the receptor, which is mediated by metalloproteases like the tumor necrosis factor alpha-converting enzyme (TACE). This licenses a second cleavage mediated by the γ-secretase complex (comprising Nicastrin, Presenilin 1/2, PEN2 (Presenilin enhancer 2), and APH1 (anterior pharynx-defective 1)) that forms the intracellular Notch receptor domain (N-ICD), which subsequently translocates into the nucleus and cooperates with the DNA-binding protein RBPJκ (recombinant binding protein suppressor of hairless; also known as CSL). This allows the conversion of the transcriptional repressor complex into an activator complex together with its co-activator Mastermind-like 1 (MAML1), and other specific co-activators [1, 2]. The activation of Notch signaling triggers the expression of various target genes, such as Hes and the Hes-related (HESR/HEY) family of basic helix-loop-helix transcription repressors [3] and subsequently regulates the expression of other genes. There is a growing appreciation that this pathway can also be orchestrated in a non-canonical manner, via HES- or RBPJk-HES-independent axis or by other means [4].

## First Evidences of Notch Signaling in the Regulation of HSPC Biology

The blood is one of the most highly regenerative tissues with an average of 10^11^–10^12^ new blood cells being produced daily in a healthy adult human. With such a high turnover system, a very fine-tuned production of cells is required to maintain the homeostasis of the blood system. Studies on signaling pathways that govern hematopoietic stem cell (HSC) self-renewal and fate decisions are critical for this balance and some of the HSC regulators have been identified including cytokines, growth factors, transcription factors, cell cycle regulators, chromatin modifiers, etc. However, the molecular mechanisms in particular the cell-to-cell interactions that support and regulate HSCs in their microenvironment (niche) are still largely unexplored [5, 6]. The evolutionarily conserved Notch signaling pathway functions as a major mediator of cell fate determination during development and also regulates diverse cellular functions including differentiation, proliferation, and survival. In hematopoiesis, the importance of Notch signaling has been associated with hematological malignancies such as T cell acute lymphoblastic leukemia [7, 8]. Differently from a conventional signaling pathway that involves the interaction between soluble factors and their receptors, Notch signaling pathway has both ligand and receptors as transmembrane proteins expressed on the cell surface. As such, Notch signaling is in many ways an ideal candidate pathway for instructing communication between HSCs and their niche, as it requires cell-to-cell contact for activation.

While known to be required during the development of the hematopoietic system [9] and in T cell development [10], whether Notch signaling plays a role in the regulation of adult hematopoietic stem cells in physiological conditions remains a subject of active debate mainly due to conflicting results obtained in several studies. First, Notch ligands and receptors have been detected in the bone marrow niche [11] and in HSPCs, respectively, both in mice and humans [12, 13]. Then, when transducing the constitutively active form of Notch1-intracellular domain (N1-ICD) [14] or the Notch target *Hes1* [15] results is an increase in the self-renewal capacity of HSPCs. Further to this, activation of Notch signaling through exposure of primitive hematopoietic cells to Notch ligands in vitro such as Jagged1 [11], extracellular Delta-like1 ligand [16], and soluble Jagged1 [17] also promotes stem cell self-renewal and inhibits differentiation.

Based on these early reports, it is therefore tempting to suggest that Notch signaling might play a role in hematopoiesis. Nonetheless, this effect is likely to be the result of super-physiological levels of Notch signaling in vitro. On the other hand, in an interesting study, it was reported that the loss of *Itch* (an E3 ubiquitin ligase which negatively regulates Notch signaling by controlling Notch receptor degradation) led to sustained levels of Notch1-mediated signaling, thus, functioning as an indirect Notch gain-of-function approach [18]. As a result, mice that were transplanted with Itch-deficient (Lin^−^Sca1^+^cKit^+^) LSK HSPCs had an expanded stem cell pool with enhanced hematopoietic contribution up to 24 weeks as compared to control LSK cells.

Despite these positive results that paved the way for approaches that allowed the development of in vitro HSPCs expansion protocols [19, 20], the understanding of the functional role of Notch signaling remains unclear as loss of function approaches have led to the proposal that the Notch pathway is not required in steady state hematopoiesis, as highlighted below (Table 1), in contrast to the in vitro studies.

**Table 1:** Evidences of Notch signaling in HSPC regulation

Study	Method	Species	Effect on general hematopoiesis	Effect on HSCs/HSPCs	Reference
Notch1 loss of function	Mx-Cre	Mouse	Impaired T cell development; myeloid and B cell development is normal	ND	[10]
RBPJk loss of function	Mx-Cre	Mouse	Impaired T cell development; myeloid and B cell development is normal	ND	[21]
Notch inhibition (dnRBPJk transduced LSK cells)	Transduction LSK cells with dnMAML1 or dnXSu(H)	Mouse	Accelerated differentiation towards B and myeloid cell lineages	Depletion of LT-HSC	[12]
Jagged1 loss of function Jagged1/Notch1 loss of function	Mx-Cre	Mouse	No effect on progenitor nor mature lymphoid, myeloid, and erythroid lineages	Normal LSK function and numbers	[22]
RBPJk loss of function	Mx-Cre	Mouse	Impaired T cell development; myeloid and B cell development is normal	Normal HSC function and numbers	[23]
Notch1 and 2 loss of function	Mx-Cre	Mouse	Myeloid and B cell development is normal in steady-state	Normal HSC function and numbers	[24]
dnMAML1 transduced HSCs	Transduction CB cells with dnMALM1	Human	Impaired T cell development; myeloid and B cell development is normal	Reduction in frequency but increase in HSC numbers	[25••]
Notch inhibition (γ-secretase inhibitor)	DAPT (γ-secretase inhibitor) administration in vivo	Human	Early differentiation of HSPCs	Higher engraftment capacity (increased generation of CD34+CD38− from CD34− HSPCs)	[26••]

*ND* not determined

While early loss of mouse *Notch1* [10] and *Rbpj* [21] studies have established the Notch1-RBPJk-HES1 axis as a major regulator of T cell differentiation, no myeloid and B cell lineage defects were observed. Although these studies did not focus on HSCs, Duncan and colleagues showed that LSK cells from a transgenic Notch reporter (TNR-GFP) mouse have high Notch activity as evidenced in bone marrow sections by co-localization of c-kit^+^ and GFP^+^ cells and by flow cytometric analysis of primitive LSK cells showing GFP expression. Interestingly, transplanting murine lineage negative (Lin^−^) cells transduced with dominant-negative of Xenopus suppressor of Xenopus *Rbpj* homologue (dnXSu(H)) resulted in the depletion of the HSC population after long-term reconstitution compared to control transduced cells [12].

Which Notch ligand/receptor pairing is preferentially used by HSPCs to signal Notch still remains elusive. Jagged1 is expressed in the bone marrow niche cells, in particular on osteoblasts [11, 27••]. Yet, loss of both *Jagged1* and *Notch1* in the bone marrow had no effect on HSC homeostasis, on hematopoietic reconstitution after genotoxic insult, or in the number of cells of different stem and progenitor subsets after bone marrow transplant thus excluding an essential role for Jagged1-mediated Notch1 signaling on HSC self-renewal or differentiation [22]. However, these studies did not rule out the possible redundant effects from other Notch receptors or ligands. Indeed, Varnum-Finney and colleagues have demonstrated that Notch2 but not Notch1 mediates LSK cell self-renewal, repopulation following transplantation and inhibits myeloid differentiation. Yet, neither loss of Notch1 nor Notch2 had any effect on the number of HSCs in steady-state conditions [24].

Perhaps more convincingly is a study undertaking a pan-inhibition of all canonical Notch signaling approach mediated by the overexpression of a dominant negative form of Mastermind-like 1 (dnMAML1). Using this approach, no effect on LSK cell function was reported with comparable repopulation capacities as to control [23]. The authors have proposed that LSK cells are exposed to very low levels of Notch signaling in vivo, contrasting to the observations reported by Duncan et al. This could explain the reason why the loss of canonical Notch does not result in dramatic effects in vivo and could also justify why in different studies, exposing cells to super-physiological levels of ligands or NICD in vitro resulted in significant increase in stem cell self-renewal.

Beyond the divergent data in mice, only recently some light on the role of Notch pathway in human HSCs in vivo has been shed. Benveniste et al. directly compared the impact of canonical Notch pathway on human purified HSCs in vitro and in vivo using the same strategy in both contexts [25••]. By overexpressing dnMAML1, the authors showed that the contribution to all the hematopoietic lineages in vivo in a xenogeneic model was comparable in a competition assay between control- and dnMAML1-HSCs, despite complete blockage of T-lineage development. Differently, the overexpression of the dnMAML1 blocked both T cell development and HSC maintenance/expansion in vitro. Although the authors appear to claim similar outcomes to the murine system [23], a close inspection of the authors’ results could infer a different interpretation. First, the percentage of dnMAML1-HSC population within the human graft was half as compared to controls. Second, the total number of dnMAML1 engrafted cells was more than double compared to controls; hence, the authors concluded that the total number of engrafted dnMAML1-HSC was unchanged relative to control HSCs [25••]. However, it can be inferred that it did as the frequency of dnMAML1-HSC was reduced with concomitant increase in total differentiated human cells.

Furthermore, the authors did not consider the possibility of non-canonical functions of the Notch receptors as a compensatory mechanism. Indeed, Delta-like1 ligand can rewire interleukin-6-mediated signal in human CD34^+^ cells through as yet unknown non-canonical mechanisms [28]. Also, they did not study the Notch pathway in human CD34^−^ HSCs or consider potential non-cell-autonomous effects that could play a role when Notch signaling is not only inhibited in HSCs. Indeed, at the top of the human hematopoietic hierarchy resides a quiescent and immature population, defined as Lin^−^CD34^−^CD38^−^CD93^hi^ (−/−/+), that has higher Notch activity as compared to the more mature but still enriched CD34^+^ HSCs population (Lin^−^CD34^+^CD45RA^−^CD90^+^CD49f^+^). The engraftment potential of the −/−/+ HSCs increased after administration of the γ-secretase inhibitor DAPT (a Notch pathway inhibitor) to the murine host in a xenograft model [26••]. Although more prolonged DAPT administration further increased human engraftment, this occurred at the expense of the CD34^+^ cells that are normally derived from the −/−/+ HSCs. These results suggest that the activation of the Notch pathway is important for the maintenance of the human HSCs repopulation capacity. However, the potential effects induced by the microenvironment were not determined since DAPT treatment may have also affected the niche cells.

In summary, these discrepancies underline the necessity for having more stringent and well-described methods to disrupt Notch signaling in vivo, but they also highlight a potential difference between the murine and the human systems.

## Loss of Notch Signaling and Myeloproliferative Disease

In contrast to the above results, several studies using mouse models, in which different members of the Notch signaling pathway have been deleted, have demonstrated that defective Notch in the bone marrow microenvironment frequently leads to the development of a myeloproliferative disease (MPD) phenotype (Table 2).

**Table 2:** Notch signaling and MPD: involvement of the microenvironment

Mouse model	Phenotype	Effect on stem cells	Reference
Psen1^+/−^/Psen2^−/−^	MPD (expanded granulocytes)	Normal side population	[29]
MMTV-Cre/Mib1^f/f^ Mx1-Cre/Mib1^f/f^	MPD (expanded granulocytes)	Expanded LSK	[30]
Fx^−/−^	MPD	Normal LSK	[31]
Mx1-Cre/Pofut^f/f^	Increased neutrophils	ND	[32]
Mx1-Cre/Adam10^f/f^	MPD (expanded)	Expanded LSK	[33]
Mx1-Cre/Ncstn^f/f^	CMML-like	Expanded LSK	[34]
Mx1-Cre/Rbpj^f/f^ Tie2-Cre/Rbpj^f/f^	MPD	Expanded LSK	[35••]

*ND* not determined

One of the earliest reports has shown that Presenilin1 haplo- and Presenilin2-deficient (*Psen1* ^+/−^ *Psen2* ^−/−^) mice have an expanded granulocytic compartment, and the animals develop signs of MPD [29]. Notch receptors are subjected to a great amount of post-translational modifications. Of those, O-fucose residues added to the receptors by the FX (Factor 10; homolog of human GDP-L-fucose synthase) enzyme are a substrate for further modification by Fringe proteins. FX mediates the addition of N-acetylglucosamine residues [36], which are essential for proper interaction with ligands [37]. *Fx* ^−/−^ mice develop a fucosylation-dependent myeloproliferative phenotype, with expanded Gr1^+^ granulocytes and Ter119^+^ erythrocytes [31]. Additionally, these mutant mice have increased common myeloid progenitor (CMP), granulocyte-macrophage progenitor (GMP), and megakaryocyte-erythrocyte progenitor (MEP) compartments, while the common lymphoid progenitors (CLPs) are significantly reduced [31]. Similarly to FX-deficient cells, *Pofut* (protein O-fucosyltransferase)-deficient marrow progenitors have defective O-fucosylation of Notch receptors and have no ability to bind to Delta-like ligands. *Pofut*-deficient mice have increased numbers of neutrophils and reduced lymphocytes. However, retrovirally transducing *Pofut*-deficient cells with N1-ICD can rescue the T cell development impairment and the Gr1^+^ cell expansion, suggesting a role for O-fucose-dependent Notch1 activity [32]. Interestingly, these observations suggest that Notch must be important at an earlier stage, perhaps at the multipotent progenitor stage, where the fine-tuning of the pathway regulates the lymphoid versus myeloid cell fate decisions.

In addition, using two Cre lines, MMTV-Cre and Mx1-Cre, Kim and colleagues showed that the loss of Mindbomb-1 (*Mib1*), an essential component for Notch ligands endocytosis, led to MPD originated from the LSK population [30]. However, transplantation of bone marrow cells from TNR reporter mice showed that LSKs from reconstituted Mib1-mutant mice had the same level of Notch activation as wild-type cells, showing that the MPD is not caused by defective Notch receptor cleavage in LSK cells. In fact, the constitutive expression of N1-ICD in *Mib1*-null microenvironment significantly delayed the development of MPD, suggesting that the defective Notch signaling between the microenvironment cells caused the MPD phenotype. Nevertheless, this data suggests that appropriate Notch signaling in the niche cells has an impact on LSK biology.

Another example relates to *Adam10* deletion in the bone marrow compartment, which leads to both cell-autonomous and non-cell-autonomous MPD. In both cases, deregulated Notch pathway triggers granulopoiesis with an expanded HSC pool [33]. These results suggest that proper Notch signaling must be maintained not only in HSPCs but also in the bone marrow microenvironment. This entails that Notch signaling is most likely important in the crosstalk between hematopoietic and niche cells, whereby the loss of proper Notch signaling in non-hematopoietic cells results in deregulated stem and progenitor cell self-renewal and differentiation. An interesting example is a skin-specific inactivation of *Notch1* and *Notch2*, or *Rbpj*, which results in a myeloproliferative disorder with increased immature myeloid cells in the bone marrow and spleen. This was caused by non-cell autonomous microenvironmental changes in which the loss of Notch triggered the production of thymic stromal lymphopoietin that led to increased levels of G-CSF resulting in non-cell-autonomous MPD [38].

Subsequently, an important work from Aifantis’s group revealed that mice transplanted with Nicastrin-deficient HSCs develop a human chronic myelomonocytic leukemia-like disease with an expanded LSK compartment. This is one of the few studies that attempted to understand the mechanisms behind this effect, with the demonstration that Nicastrin-deficient LSK cells showed a derepressed myeloid program, which is thought to be carried out by HES1-mediated inhibition of *Cebpa* and *Spi1* expressions [34].

Finally, an elegant study from the Carlesso laboratory showed that conditional deletion of *Rbpj* using Mx1-Cre leads to myeloproliferative disease [35••]. When performing reciprocal transplantation experiments, the authors observed that the mutant-LSK pool was significantly increased but the GMP, CMP, and MEP subsets were unaffected in wild-type mice but without signs of splenomegaly and with similar survival as the control cohort. In contrast, when transplanting wild-type HSPCs into mutant mice, CMPs and GMPs in the bone marrow and spleen were significantly increased similarly to the parental *Rbpj* ^−/−^ mice, resulting in lethal MPD. Interestingly, the disease evolved faster after bone marrow transplant. Additionally, the wild-type LSK pool in *Rbpj* ^−/−^ recipients was less affected than in parental *Rbpj* ^−/−^ mice or *Rbpj* ^−/−^ cells transplanted into wild-type mice demonstrating a cell-autonomous contribution of RBPJ in HSC regulation. However, this study also demonstrates that cell autonomous loss of Notch activation is not sufficient to develop myeloid disease, while loss of Notch signaling in the microenvironment induces lethal MPD in a non-cell-autonomous manner. Mechanistically, deletion of *Rbpj* leads to increased expression of miR-155 in bone marrow niche cells where the NICD/RBPJ complex acts as a transcription repressor of miR-155. miR-155 upregulates NF-κB activation by targeting one of its inhibitors κB-Ras1, leading to increased expression of G-CSF and TNFα thus, inducing a persistent inflammatory state in the bone marrow.

In summary, it is remarkable that inhibition of Notch, through the loss of different regulators of the pathway, seems to expand the most primitive HSCs. This is probably due to early differentiation induced by the loss of a quiescent state imposed by Notch signaling. It will be important to clarify whether this effect leads to stem cell exhaustion or if in some cases, stem cells can divide seemingly indefinitely as has been described before [18]. But most importantly, these studies demonstrate that Notch activation via Notch ligand-receptor interactions between niche cells is also required for normal hematopoiesis. Altogether, these studies highlight the importance of Notch signaling in the bone marrow microenvironment for the proper regulation of hematopoiesis.

## HSC and Niche Crosstalk Via Notch Signaling

Several studies have looked for the presence and functions of Notch ligands in different bone marrow niche cells (endothelial cells, osteoblasts, and mesenchymal stromal cells) that are able to support HSPCs in vitro or appear closely associated to them in vivo.

Indeed, bone marrow endothelial cells express Notch ligands and its expression can be regulated by pro-inflammatory stimuli in vitro and in vivo [39]. In a study by Fernandez and colleagues, a human bone marrow endothelial cell line called “BMEC” and primary human bone marrow endothelial cells were found to express Jagged ligands. The capacity of these cells to expand hematopoietic progenitors in an in vitro co-culture system was dependent on Notch signaling. The authors also demonstrated that the ligand density and Notch signal intensity could result in different degrees of HSPCs expansion. Furthermore, in vivo pro-inflammatory cues (TNFα, LPS) caused an increase of Jagged2 expression on endothelial cells and Notch1 and Notch2 receptors on hematopoietic progenitors, which were associated with increased Notch activation.

Later, Butler and colleagues showed that endothelial cells are able to support long-term expansion of Notch-activated (TNR-GFP^+^) LSK HSPCs, but not Notch1 and Notch2 defective HSCs (Notch1^−/−^Notch2^−/−^CD34^−^Flt-3^−^LSK) [40]. The authors also elegantly showed that HSCs secrete vascular endothelial growth factor A (VEGF-A) when exposed to soluble stem cell factor in vitro*.* VEGF-A secreted by HSCs in turn stimulates the translocation of Jagged2 ligand on the endothelial cell surface, which then supports the expansion of the HSCs by interacting with Notch1 and Notch2 receptors. This study also demonstrated that in vivo inhibition of both VEGFR2 and VE-cadherin, essential for endothelial angiocrine signaling, resulted in a profound decrease in the instructive capacity of endothelial cells. This caused impaired reconstitution of TNR-GFP^+^ hematopoietic cells after sub-lethal irradiation due to a significant decrease in the regeneration of TNR-GFP^+^ HSCs, leading to demise of the treated mice. These results not only support the importance of the vascular niche but also suggest that during hematopoietic recovery, angiocrine expression of Notch ligands by endothelial cells is essential to balance the expansion and differentiation of HSCs and also reveal a crosstalk between HSCs and their niche elements.

In the same vein, Butler’s group showed that in vivo conditional deletion of *Jagged1* in endothelial cells (VE-cadherin-Cre) caused a decrease in the number of phenotypical and functional HSCs in steady-state, which was associated with a significant reduction in Notch activation in LSK HSPCs [41•]. In addition, mutant mice have deficient hematopoietic recovery after sub-lethal irradiation leading to reduced survival rate compared to the control group. In vitro co-cultures with endothelial cells from mutant mice resulted in greater hematopoietic expansion exclusively from differentiated cells with a significant increase in myeloid cells. These data emphasize the importance of niche Jagged ligands in balancing expansion and differentiation of HSPCs, demonstrating that Notch signaling activation by bone marrow endothelial cells through Jagged1 ligand is essential to maintain homeostatic and regenerative hematopoiesis.

Of note, this data contradicts the observations of Mancini et al. [22], in which conditional deletion of *Jagged1* driven by the Mx1 promoter had no effect on hematopoietic regulation at steady-state or regenerative conditions. Butler’s group verified that while the VE-cadherin-Cre efficiently deleted the floxed *Jagged1* in bone marrow endothelial cells, the inducible Mx1-Cre system did not. These data indicate that the observations of Mancini et al. could be associated to inefficient deletion of endothelial-specific *Jagged1*.

Apart from endothelial cells, other bone marrow stromal cells have also been shown to be associated to HSPC regulation via Notch signaling. A subset of human CD146^+^ perivascular mesenchymal stromal cells (MSCs) support long-term culture of functional human HSPCs partially through Notch activation [42]. Indeed, CD146^+^ perivascular cells express high levels of Notch ligand Jagged1. Accordingly, Notch activation was demonstrated to be significantly higher in HSPCs co-cultured with CD146^+^ cells than in co-cultures with total MSCs or CD146^−^ cells. Moreover, Notch inhibition with DAPT resulted in decreased HSPCs number and CFUs and increased B cell differentiation. These results suggest that CD146^+^ perivascular cells can also regulate self-renewal and lineage commitment of HSPCs through Notch signaling.

Interestingly, a recent study shed some light on the human bone marrow niche in vivo by analyzing human bone biopsy specimens and human-mouse xenografts. This study revealed that HSCs with superior regenerative and self-renewal capacity tend to localize to endosteal regions of the trabecular bone area (TBA) [27••]. These HSCs have distinct molecular activation enriched with Notch signaling signature compared to HSCs localizing in the long bone area (LBA). Particularly within the TBA, phenotypic human HSCs and HPCs preferentially locate in the endosteal over vascular regions. The authors also showed that osteoblasts from the TBA have increased expression of Notch ligands Jagged1, Jagged2, and Delta-like4 compared to osteoblasts from the LBA, consistent with the HSC activation profile. In particular, a threefold higher proportion of osteoblasts expressing Jagged1 was detected in the TBA compared to LBA. Additionally, Jagged1-binding HSPCs were found to have higher CFU capacity and repopulating capacity. Upon Notch inhibition with DAPT treatment in vivo, total human engraftment and HSPC numbers were not affected in the LBA, whereas there was a significant reduction in the TBA, suggesting that only this region is sensitive to Notch inhibition leading to an overall reduction in human engraftment in a region-specific manner. These observations support a critical role of Notch signaling in HSC regulation through interactions with specific bone marrow niche cells.

In a subsequent study, further investigation on the role of endosteal osteoblasts in HSC regulation showed that osteoblast ablation (Col2.3Δtk) leads to reduced quiescence, long-term engraftment, and self-renewal capacity of HSCs [43]. Moreover, osteoblast ablation in a transgenic chronic myeloid leukemia mouse model showed accelerated leukemia progression with reduced survival compared to control mice. In this mouse model, osteoblasts were found to express high levels of Jagged1 and co-culture of leukemic or normal HSCs with stromal cells expressing Jagged1 resulted in reduced cell cycling and total number of cells. These data suggest that Jagged1 signaling in the osteogenic niche is essential for maintaining HSC quiescence and long-term self-renewal capacity.

Finally, as further evidence of the importance of Notch ligands in the osteogenic niche, in humanized mice expressing human Jagged1 in osteoblasts, human HSPC repopulation is higher than in control mice [44]. In addition, mice with human Delta-like1-expressing osteoblasts develop severe osteosclerosis, which suppresses the generation of human B lymphocytes and also retention of human HSPCs that is likely due to the severe osteosclerosis in the bone marrow [45].

Together, these studies focusing on the osteogenic niche suggest a role for Notch signaling through osteoblasts on regulation of HSC self-renewal and quiescence, particularly through Jagged1 in the trabecular bone area, where interactions and Notch activation seem to be higher. However, the activated Notch receptors and the molecular mechanisms behind this regulation are still not understood. Also, a more comprehensive study is necessary to clearly identify the functions of the different ligands on HSPC regulation both in vitro and in vivo.

## Conclusions

Although several authors have suggested Notch signaling as dispensable in the adult hematopoietic pathway using different genetic approaches, the maintenance of adequate dose of Notch signaling seems fundamental in order to avoid the development of hematological malignancies. As reported, different populations within the primitive hematopoietic stem and progenitor compartments seem to depend differently on Notch at different stages of differentiation. As already mentioned, it will be relevant to understand the functions of the different ligand/receptor pairings in different primitive populations. We have reviewed some studies that have shed light on the mechanisms by which Notch regulate HSPCs, and will certainly learn more about the fine-tuning of this signaling pathway as soon as more stringent and well-described methods to inhibit and activate Notch become available.
